# Studying Erythromelalgia Using Doppler Flowmetry Perfusion Signals and Wavelet Analysis—An Exploratory Study

**DOI:** 10.3390/biomedicines11123327

**Published:** 2023-12-16

**Authors:** Luis Monteiro Rodrigues, Joana Caetano, Sergio Faloni Andrade, Clemente Rocha, José Delgado Alves, Hugo Alexandre Ferreira

**Affiliations:** 1 CBIOS—Research Center for Biosciences and Health Technologies, Universidade Lusófona Lisboa, 1700-097 Lisbon, Portugal; joana.r.caetano@hff.min-saude.pt (J.C.); sergio.andrade@ulusofona.pt (S.F.A.); c.rocha.dr@gmail.com (C.R.); 2 Immuno-Mediated Systemic Diseases, Medicina IV, Hospital Fernando Fonseca, 2720-276 Amadora, Portugal; jose.alves@nms.unl.pt; 3 Nova Medical School, Nova University of Lisboa, 1169-056 Lisboa, Portugal; 4 Faculty of Sciences, Institute of Biophysics and Biomedical Engineering, University of Lisbon, Campo Grande, 1749-019 Lisboa, Portugal; hhferreira@fc.ul.pt

**Keywords:** erythromelalgia, skin perfusion, laser doppler flowmetry, wavelet analysis

## Abstract

Erythromelalgia (EM) is a rare disease, which is still poorly characterized. In the present paper, we compared the hand perfusion of one female EM patient, under challenges, with a healthy control group. Using a laser Doppler flowmeter (LDF) with an integrated thermal probe, measurements were taken in both hands at rest (Phase I) and after two separate challenges—post-occlusive hyperemia (PORH) in one arm (A) and reduction of skin temperature (cooling) with ice in one hand (B) (Phase II). The final measurement periods corresponded to recovery (Phases III and IV). The control group involved ten healthy women (27.3 ± 7.9 years old). A second set of measurements was taken in the EM patient one month after beginning a new therapeutic approach with beta-blockers (6.25 mg carvedilol twice daily). Z-scores of the patient’s LDF and temperature fluctuations compared to the control group were assessed using the Wavelet transform (WT) analysis. Here, fluctuations with |Z| > 1.96 were considered significantly different from healthy values, whereas positive or negative Z values indicated higher or lower deviations from the control mean values. Cooling elicited more measurable changes in LDF and temperature fluctuations, especially in higher frequency components (cardiac, respiratory, and myogenic), whereas PORH notably evoked changes in lower frequency components (myogenic, autonomic, and endothelial). No significant Z-score deviations were observed in the second measurement, which might signify a stabilization of the patient’s distal perfusion following the new therapeutic approach. This analysis involving one EM patient, while clearly exploratory, has shown significant deviations in WT-derived physiological components’ values in comparison with the healthy group, confirming the interest in using cold temperature as a challenger. The apparent agreement achieved with the clinical evaluation opens the possibility of expanding this approach to other patients and pathologies in vascular medicine.

## 1. Introduction

Erythromelalgia (EM), or Mitchell’s disease, is a rare condition affecting up to 2 in 100,000 patients in Europe each year [1,2]. Patients display a wide variety of clinical manifestations and severity, with acute burning pain, erythema, and discomfort, especially at the extremities [1,2,3]. Primary and idiopathic EM can be present at any age, although it is more common in the first decade of life, while secondary EM is more common in older adults and is primarily associated with other comorbidities [1]. EM is still a poorly known disease, such that its diagnosis and treatment remain challenging [1,4,5]. A few recently published reviews clearly indicate that much more clinical and applied research is urgently needed to better control and understand the disease’s course [5,6,7].

EM pathogenesis has been associated with a variety of causes with discrete histopathology changes, including arteriovenous (AV) shunting, but these associations are insufficient to explain the microcirculatory functional changes described [8,9,10,11,12,13]. Nonspecific capillary proliferation and vasculopathy have been suggested to evoke skin hypoxia in erythromelalgia [14], while another recent study proposed an association between autonomic and vascular dysfunction as capable of reducing skin perfusion [6,15]. It was also observed that in patients with primary EM, local heat provocation raised the skin’s perfusion as measured with laser Doppler flowmetry (LDF) and the temperature of the plantar region of the foot (location of multiple AV shunts), while decreasing in the dorsum of the foot [12]. 

Our experience studying the microcirculation physiology of human extremities might offer a different perspective to approach and explore these phenomena [16,17,18,19,20]. For that purpose, we measured the perfusion responses to suprasystolic pressure (post-occlusive reactive hyperemia, PORH) and to controlled cold temperature for an EM patient, and compared them with a healthy cohort. All responses were registered with LDF technology and then “decomposed” with the Wavelet transform. Our study objective was to detail the typical oscillatory non-stationary LDF records, also known as flowmotion, that reflect contributions from the main factors (cardiac, respiratory, myogenic, autonomic, and endothelial) driving perfusion [21]. Through this analysis, we also expected to determine the specific component changes that might improve our understanding of EM pathophysiology. 

## 2. Methods

### 2.1. The Patient

A 35-year-old Caucasian woman without any other significant medical records showed a consistent 15-year history of bilateral palmar warmth, erythema, and burning pain, extending from the fingers into the entire hand. This patient was selected from the specialized medicine consultation at the Immuno-mediated Systemic Diseases (Hospital Fernando Fonseca, Amadora, Portugal). In the four years prior, she reported the same symptoms in both feet, worsening over that time. These symptoms were persistent, also occurring at rest, and were typically exacerbated by physical activity or by any increase in ambient heat. Physical examination depicted symmetrical erythema and warmth of the palms, including fingers, and soles.

Various complementary tests were conducted, including laboratory work and electromyography, showing no relevant findings and no evidence of peripheral neuropathy. Mutation of the gene *SCN9A* was negative. Nailfold capillaroscopy revealed a decreased capillary density (6/mm), dilated and giant capillaries (largest capillary diameter of 68 μm), and no hemorrhages or abnormally shaped capillaries. After her idiopathic EM diagnosis, the patient started oral misoprostol (0.4 mg) twice a day, and pregabalin (50 mg) once a day, for two months. A clear improvement in the patient’s condition was registered after the five days of intravenous iloprost (50 mcg per day for five days, two weeks prior to the functional assessment), visible by a decrease in redness of her hands and fingers, but her main discomfort persisted.

### 2.2. The Control Group

A convenience group of ten healthy women (27.3 ± 7.9 years old) was selected as the control group after informed written consent. Specific inclusion/non-inclusion criteria previously defined for similar research [16] were applied, with all participants normotensive with no signs of vascular impairment, confirmed using the ankle–brachial index (ABI of 1.1 ± 0.1) [22] and normal body mass index (BMI of 24.6 ± 1.8 kg/m^2^). All were non-smokers and free of any regular medication or food supplementation. Participants were asked to refrain from consuming caffeinated and/or any other vasoactive beverages for 24 h prior to the experiments. Table 1 summarizes the characteristics of participants at inclusion.

All procedures respected all principles of good clinical practice in accordance with the Declaration of Helsinki and respective amendments [23], being previously approved by the institutional Ethical Commission.

### 2.3. Experimental

Measurements took place once, on inclusion day, in a room with controlled temperature (21 ± 1 °C), humidity (40–60%), and light after acclimatization to these conditions (approximately 20 to 30 min while lying comfortably seated). All baseline variables were continuously recorded (Phase 1) until full stabilization where the final three minutes were used for calculation purposes.

**Figure 1:**
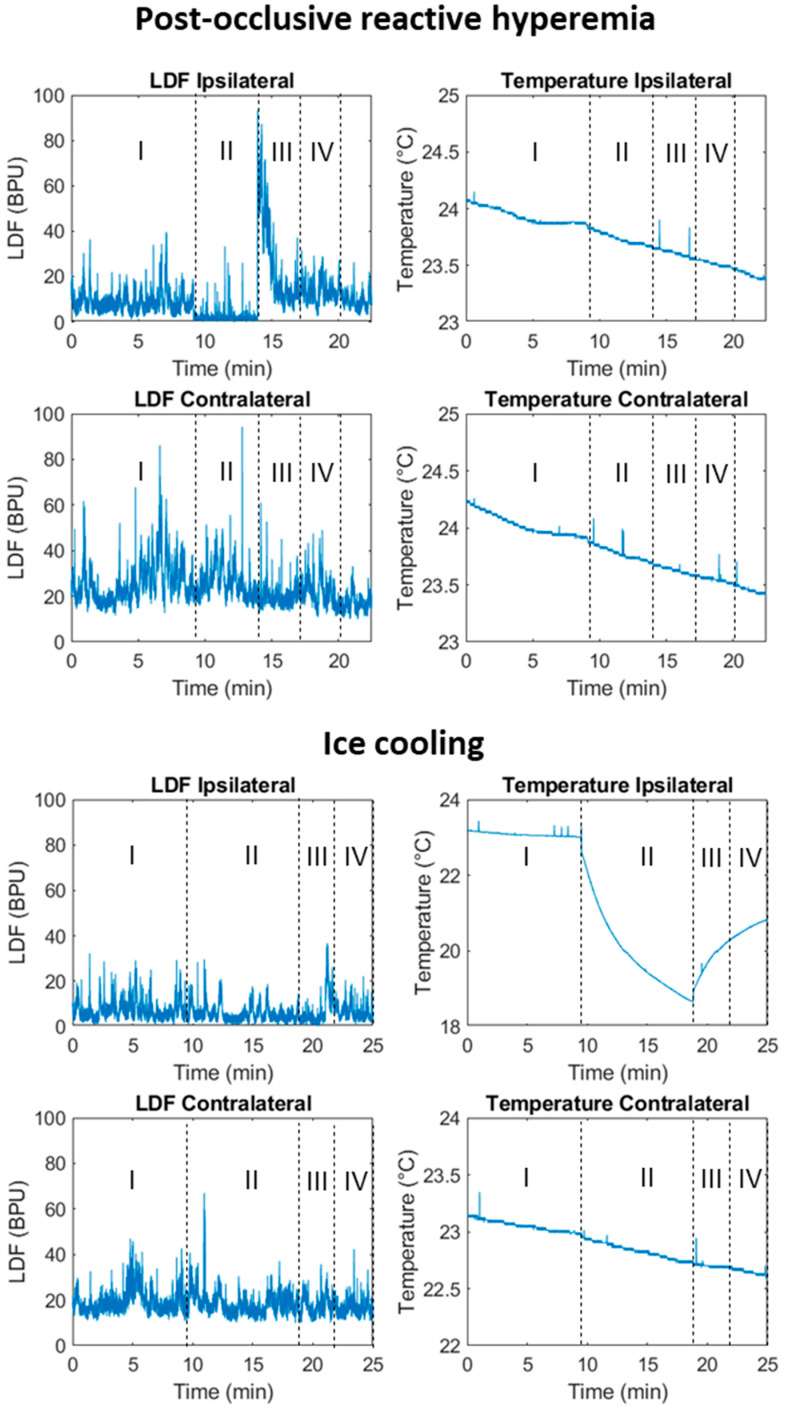
Illustrative example of laser Doppler flowmetry (LDF) and skin temperature signals in a healthy participant, obtained during the post-occlusive reactive hyperemia (PORH, **top** panel) and during cooling (**bottom** panel). The different phases of the challenges are represented: I, baseline; II, challenge with PORH or with cooling; III, early recovery, during the 3 first minutes after the challenge; and IV; late recovery, during the last 3 min of recovery. (BPU. Blood Perfusion Units).

All participants were submitted to two sequential challenges—reactive hyperemia and the reduction of skin temperature by contact with ice (Figure 1). Challenges were applied to only one randomly chosen upper limb. The order of the challenges was randomly chosen, and the sequence was separated by a 30 min washout interval between measurements.

### 2.4. Post-Occlusive Reactive Hyperemia (PORH)

The PORH maneuver was applied in accordance with established guidance, using a pressure cuff on the randomly chosen arm [24,25]. After perfusion stabilization (Phase I), the cuff was rapidly inflated with 200 mmHg to occlude the brachial artery for approximately 5 min to ensure hemodynamical stabilization in the area (Phase II). The cuff was then deflated for recovery, allowing for registration at the beginning of an early recovery phase, between 0 and 3 min (Phase III), and late recovery between 3 and 6 min (Phase IV).

### 2.5. Cooling

Skin cooling is not commonly used to explore cardiovascular and hemodynamical physiology. Therefore, we adapted our protocol to that purpose using recently published work [26]. The palms of both hands faced down on a pad, with one resting on an ice-frozen platform covered with a cotton cloth. Perfusion and temperature were continuously monitored in both hands before cooling (Phase I), during cooling (Phase II) for ten minutes, during early recovery (Phase III) (early recovery) between 0 and 3 min, and late recovery between 3 and 6 min (Phase IV) (late recovery).

The patient was again tested with the same experimental procedure thirty days after beginning a new therapeutic prescription with a beta-blocker (6.25 mg carvedilol twice daily) (see ahead). 

### 2.6. Measurement and Data Analysis

Blood perfusion was continuously assessed through LDF (Perimed PF5010, Perimed, Järfälla, Sweden, with a pair of P457 probes secured with PF105–3 tape and using a sampling rate of 32 Hz) [21,27,28]. LDF sensors (perfusion and temperature) were applied in the ventral aspects of the third finger, in both hands. This strategy substantially reduces variability when measuring in distal areas [16,29]. The LDF signal was expressed in arbitrary Blood Perfusion Units (BPUs). 

Skin perfusion changes were also followed by a non-contact polarized light spectroscopy (PSp) system (Tissue Viability Image System—TiVi701; WheelsBridge, Linköping, Sweden). The system used an adapted digital camera placed 30-60 cm above the dorsal aspect of both hands. The PSp assesses the concentration of red blood cells (CRBC, in arbitrary units) in real-time through video images of the microcirculation obtained with cross polarization filters in the selected region of interest (ROI) [30,31]. 

To look deeper into LDF flowmotion, we analyzed changes occurring under the challenges by the wavelet transform (WT), a well-known analytical instrument mostly applied to LDF “de-noising” and analysis refinement [32]. These oscillations explain flowmotion and illustrate the influence of heart rate, respiration, myogenic, autonomical, and endothelial (NO-dependent and independent) vascular smooth muscle relaxation [26,30]. Herein, the Wavelet Coherence Toolbox (morlet wavelet) [33] was used together with in-house developed MATLAB (v. R2021b Mathworks, Natick, MA, USA) scripts to “decompose” the LDF signal into frequency bands corresponding to endothelial [0.0095; 0.021] Hz, autonomic [0.021; 0.052] Hz, myogenic [0.052; 0.15] Hz, respiratory [0.15; 0.6] Hz, and cardiac [0.6; 2] Hz activities.

Hypothesizing a relationship between LDF and temperature signals, the same WT analysis was also applied to temperature signals, for the first time.

The blood pressure (systolic, diastolic and mean) was also measured in the arm (Tensoval Comfort, Hartman, Unna, Germany) in each procedural phase.

Descriptive and comparative statistical analysis was performed using the IBM SPSS version 28.0 software (IBM Corp., Armonk, NY, USA). Median values of LDF and temperature signals at the different frequency bands were computed for each phase. Herein, the last 3 min before provocation and the middle 3 min of the provocation were considered for Phases I and II, respectively, as well as the full Phase III and Phase IV durations (3 min each). Additionally, relative signals for each frequency band, $S_{rel}^{freq}$, were computed as follows:

(1) $$S_{rel}^{freq}=\frac{S^{freq}\times 100\%}{S^{total}}$$

in which $S^{freq}$ is the mean LDF or temperature wavelet component amplitude at the frequency band (*freq*), corresponding to endothelial, autonomic, myogenic, respiratory, or cardiac activities, and $S^{total}$, corresponds to the overall mean LDF or temperature wavelet amplitude comprising all the frequency bands referred.

Intra-individual perfusion signal variations between hands were tested using Wilcoxon’s test for repeated measurements with non-normal distributions in the control group. Friedman’s test with Bonferroni correction was then used to compare the different phases in the referred group. Differences in the patient relative to the control group at baseline and after one month of a new medication with beta-blockers were assessed using Z-scores. A confidence level of 95% (*p* < 0.05) was adopted.

## 3. Results

Illustrative examples of LDF and temperature responses to the PORH and ice cooling challenges are show in Figure 1 in a healthy participant.

Different responses can be observed for LDF and temperature signals depending on the challenge. With the PORH challenge, the LDF signal amplitude is greatly reduced during cuff inflation, especially in the challenged limb (ipsilateral). A similar effect is noted in the (contralateral) non-challenged limb. After cuff deflation, a significant overshoot resulted (Figure 1, top panel, ipsilateral side). The ice cooling challenge shows a significant decrease in skin temperature in both hands during ice application (to the single hand), followed by an increase in temperature after the application had ended (Figure 1, bottom panel).

Additionally, median and interquartile range values of nominal LDF and temperature signals are shown in Table 2 for the healthy control group, and for both ipsilateral and contralateral sides of the challengers.

No significant differences in global LDF and temperature values were observed between limbs ipsilateral and contralateral to the PORH application side for each phase. Conversely, significant differences between phases were observed for ipsilateral LDF signals (Friedman’s test, *p* = 0.022; pair-wise Wilcoxon’s test, Phases II–III, *p* = 0.021 Bonferroni-corrected).

Moreover, no significant differences in global LDF were observed between limbs ipsilateral and contralateral to the ice cooling application side for each phase. Nonetheless, significant differences in global temperature signals were observed between limbs for Phases II (*p* = 0.047), III (*p* = 0.028) and IV (*p* = 0.028), corresponding to the challenge per se and the early and late recovery phases, respectively.

Significant differences between phases were observed for ipsilateral LDF signals (Friedman’s test, *p* < 0.001; pair-wise Wilcoxon’s tests, Phases I–II, *p* = 0.019, I–III *p* = 0.034, I–III, *p* < 0.001), contralateral LDF signals (Friedman’s test, *p* = 0.012; pair-wise Wilcoxon’s test, Phases I–IV, *p* = 0.006), ipsilateral temperature signals (Friedmann test, *p* < 0.001; pair-wise Wilcoxon tests, Phases I–II, *p* = 0.006, I–III *p* < 0.001, I–IV, *p* = 0.019), and contralateral temperature signals (Friedman’s test, *p* = 0.004; pair-wise Wilcoxon’s tests, Phases I–III *p* = 0.034, I–IV, *p* = 0.003) in the ice cooling challenge. 

**Figure 2:**
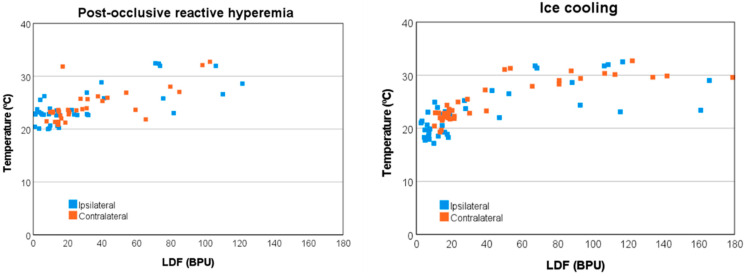
Temperature vs. laser Doppler flowmetry (LDF) signals of healthy participants for the ipsilateral and contralateral limbs obtained during the post-occlusive reactive hyperemia (PORH) (**left**) and during ice cooling (**right**). (BPU. Blood Perfusion Units).

Finally, both LDF and temperature signals were observed to be correlated across phases and limbs, showing a larger overall Spearman’s correlation, *r_s_* = 0.820 (*p* < 0.001) for the ice cooling challenge than for the PORH challenge *r_s_* = 0.633 (*p* < 0.001) (Figure 2).

When considering relative signal components of LDF and temperature signals, namely cardiac, respiratory, myogenic, autonomic, and endothelial activities, significant differences were observed between limbs. For the PORH challenge, LDF differences were observed in cardiac and autonomic components during Phase II (the challenge itself, *p* = 0.005, and *p* = 0.009, respectively), and during Phase III (early recovery) for the autonomic component alone (*p* = 0.009). For the ice cooling challenge, the most notable significant difference was observed in the temperature signals’ myogenic component for Phase III (*p* = 0.007). Furthermore, for the PORH challenge, significant differences were observed only for LDF signals across the different phases, namely regarding the ipsilateral limb endothelial (*p* = 0.001), autonomic (*p* < 0.001) and myogenic (*p* = 0.015) activities, and also for the contralateral limb regarding respiratory (*p* = 0.020), and cardiac (*p* = 0.040) activities. The most assumed differences for the limb ipsilateral to the challenge were observed for the autonomic and endothelial components of the LDF signal in the comparisons between Phases I (baseline rest) and II (during the challenge) (autonomic: 31.5% vs. 10.5%; endothelial: 51.1% vs. 76.4%, respectively) and between Phases II and IV (final recovery) (autonomic: 10.5% vs. 28.2%; endothelial: 76.4% vs. 47.3%, respectively). The most significant difference for the limb contralateral to the challenge was observed between Phases I and IV (2.8% vs. 3.4%) for the respiratory component of the LDF signal.

Significant differences in LDF across the different phases were observed in the ice cooling challenge only in the ipsilateral limb cardiac (*p* = 0.033) activity, comparing Phases I and III (early recovery) (1.9% vs. 2.6%). For temperature signals, both ipsilateral and contralateral significant differences were observed regarding cardiac (*p* = 0.007, and *p* = 0.038, respectively) and respiratory (*p* = 0.002, and *p* = 0.019, respectively) activities, and also for the ipsilateral limb myogenic (*p* = 0.015) activity. Once more, WT analysis shows that the PORH challenge seems to induce measurable changes in LDF signals and the ice cooling challenge in temperature signals. Interestingly, the former seems to be related more significantly to lower frequency physiological responses, i.e., endothelial, autonomic, and myogenic activities, while the latter appears to be related to higher frequency physiological responses, especially regarding the cardiac, respiratory, and myogenic activities. The contralateral limb responses in both challenges are also noticeable, especially regarding cardiac and respiratory activities.

Regarding the patient herein reported, Table 2 illustrates that during the POHR challenge, the ipsilateral LDF signal decreases in amplitude during Phase II (cuff inflation) in comparison to Phase I (baseline rest), in a more modest manner than the median of the healthy control group. Additionally, a further decrease was observed during Phase III (early recovery), opposed to the healthy control group. However, after treatment with carvedilol for 30 days, the LDF signal ‘behavior’ seems to approach the median of the healthy control group, with a larger amplitude decrease during Phase II and a considerable increase in amplitude during Phase III, followed by a rapprochement to Phase I amplitude values during Phase IV (late recovery). During the ice cooling challenge, disparate signal ‘behavior’ was also observed, especially regarding ipsilateral temperature signals. In particular, before treatment, no significant changes in temperature were observed during Phase II (ice placed in contact with the skin of the participant), unlike the healthy control group. Again, after treatment the temperature signal ‘behavior’ seems to follow closer to the healthy control group, showing a decrease in temperature during Phase II, followed by a return to baseline rest temperature values during recovery (Phases III and IV). 

**Figure 3:**
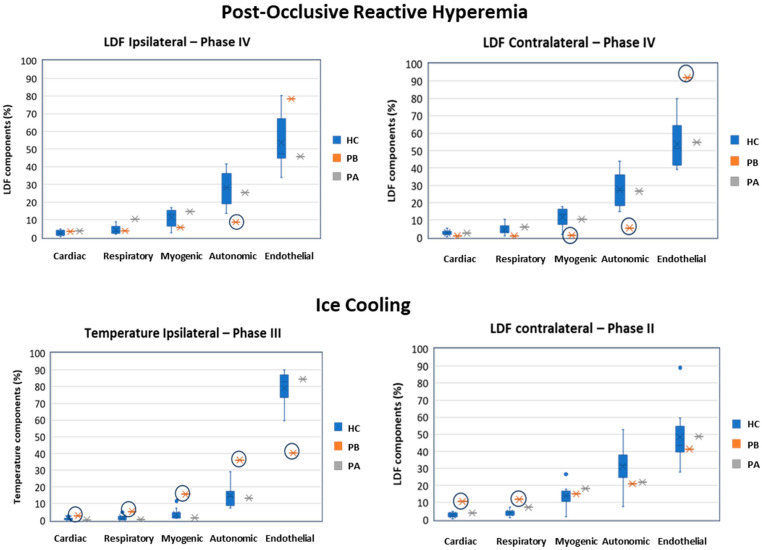
Laser Doppler flowmetry (LDF) and temperature signal components, obtained during the post-occlusive reactive hyperemia (PORH, **top** panel) and during ice cooling (**bottom** panel), and as computed with the Wavelet transform. The different phases of the challenges are represented: I, baseline; II, challenge with PORH or with ice cooling; III, early recovery, during the first 3 min after the challenge; and IV; late recovery, during the last 3 min of recovery. HC (blue). Healthy control group (*n* = 10 females); PB (orange) Patient before treatment and PA (grey) the Patient after 30 days treatment with carvedilol. Circles denote patient signals deviating Z-score > 1.96 in absolute value, depicting a significant difference from the healthy control group.

Figure 3 shows plots of LDF and temperature signal components after WT analysis (relative signal components, as computed according to Equation (1)) from which patient signal component deviations of |Z| > 1.96 from the healthy control group are highlighted. We observe that in the PORH challenge (Figure 3, top panel), the patient shows significant differences in both ipsilateral and contralateral LDF signals in low frequency physiological activities, most notably the autonomic (ipsilateral, Z = −2.00; contralateral, Z = −2.26) activity and also the myogenic (Z = −1.99) and endothelial (Z = 2.76) activities during Phase IV (late recovery). Additionally, differences were observed in the ipsilateral limb myogenic activity regarding temperature signals during Phase I (at rest, Z = 3.08) and Phase III (early recovery, Z = 2.12), in the autonomic activity regarding temperature signals during Phase I (Z = 2.03) and Phase IV (Z = 2.56), and regarding LDF signals during Phase I (Z = −2.23). Differences were also observed in the contralateral limb myogenic activity regarding LDF signals during Phase IV (Z = −1.99); and in autonomic activity regarding temperature and LDF signals during Phase IV (Z = 2.56, and Z = −2.26, respectively); and finally in endothelial activity regarding LDF signals during Phase IV (Z = 2.76).

In the ice cooling challenge (Figure 3, bottom panel), the patient shows significant differences in all ipsilateral temperature signal components during Phase III (early recovery), namely regarding cardiac (Z = 2.94), respiratory (Z = 2.68), myogenic (Z = 3.61), autonomic (Z = 3.19), and endothelial (Z = −3.63). In parallel, ipsilateral LDF signal components also showed significant differences during Phase III in the autonomic (Z = −2.26) and endothelial (Z = 2.04). After treatment with the beta-blocker, both LDF and temperature signal components were no longer significantly different from the healthy control group signals. Finally, it is also worth mentioning that contralateral LDF signal components regarding cardiac (Z = 6.11) and respiratory (Z = 4.41) showed the largest significant differences compared to the healthy control group during Phase II. Once again, after treatment, signal components were no longer significantly different compared to the healthy control group.

## 4. Discussion

As shown, both PORH and cooling challengers applied to one limb evoked similar reflex response in perfusion with distinct magnitudes in both hands. Intraindividual differences between hands were not found for these responses. Recent publications have discussed the mechanisms involved in PORH and in ice cooling maneuvers [20,34,35], however such discussions are beyond the scope of this study. The presence of a similar impact on the contralateral limb indicates that these responses are not local, but instead result from a centrally mediated adaptive response that is proportional (in intensity and duration) to the stimulus [16,20]. In our case, the cooling challenge seems to evoke more pronounced differences in both LDF and temperature signals between ipsilateral and contralateral limbs, as well as between the different phases, in comparison with the PORH challenge (Table 2). These results suggest a new interest in using cooling to study cardiovascular adaptive mechanisms, on par with other commonly used challengers such as reactive hyperemia, hypoxia or hyperoxia, and heat. 

Regarding EM research, LDF is still considered as a reference technology for the non-invasive assessment of skin microcirculation [24,36,37,38,39]. In a recent study LDF was recommended as a simple technology for sensitive detection of early-stage peripheral disease [40]. Our experience recommends some precautions when using LDF as a measuring tool for these types of studies. LDF variables are expressed in arbitrary units (BPUs), immediately alerting the absence of a direct relationship with an identifiable physiological function. The variable we register might be described as “perfusion”, rather than blood flow (volume/time). Finally, we must consider the peculiar structure of microcirculation in the skin involving two vascular plexuses (superficial and deep) with different dimensions and blood mass travelling between these structures by connecting vessels during adaptation to acute changes [41,42]. LDF is a single-point measurement optical technology, meaning that (a) it measures a very restricted area in the skin; (b) its penetration depends, among others, on the frequency of the laser used (in our case 780 nm); and (c) does not allow for a clear identification of the tissue and vessels involved. 

**Figure 4:**
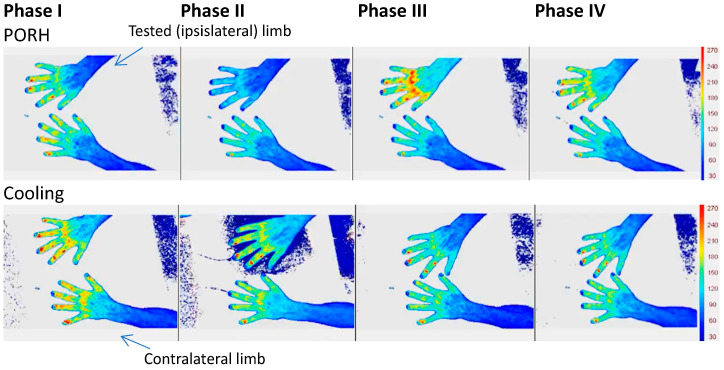
Example of a TiVi image presentation for both post occlusive reactive hyperemia (**top**) and cooling protocols (**bottom**) during all phases of the experimental procedure. In both examples, the challenge was applied in the right limb/hand (see text).

In our experimental approach, we used LDF and followed each experiment with a PSp unit to ensure that the obtained response to our challenge was consistent and attributable to the challenger. Both technologies are optical-based, detecting perfusion at different depths. By using PSp as a real-time imager, the observer could confirm the association of the PSp color pattern changes to the progress of each experiment. An illustrative example is shown in Figure 4. 

Furthermore, being aware of the significance of LDF flowmotion, well-illustrated by its non-linear, multi-scaled, oscillatory register, we decided to decompose its curves through the WT. The utility of this strategy has been demonstrated in various scenarios [43]. Herein, significant differences in perfusion were observed between limbs, namely regarding the cardiac and autonomic components of the LDF signal during the PORH challenge, something not observable using the single LDF signal.

Regarding temperature, significant differences were observed between limbs during the cooling challenge using both temperature signals and the WT-derived myogenic component of such signals, showing that WT analysis could further support in providing a physiological explanation of these findings.

The WT analysis also provided a more in-depth putative understanding of the physiological changes elicited by the challengers. PORH seems to be related to endothelial, autonomic and myogenic activities, whilst cooling appears to be related to cardiac, respiratory, and myogenic activities, as observed in the ipsilateral limbs. Additionally, changes were observed in the contralateral limbs, namely regarding cardiac and respiratory components, which again supports a more centrally mediated response between limbs, impacting both perfusion and temperature signals through specific mechanisms yet to be elucidated (Figure 1 and Figure 2).

Our analysis further comprised assessing Z-scores of the patient’s LDF components and temperature fluctuations in comparison to the control group. The Z-score enables the assessment of the distance between a given point and a normal or Gaussian distribution characterized by the mean and standard deviation. In our case, it made it possible to see how distant—or not—the values of a given metric evaluated in the patient were from the distribution of that metric in a healthy (control) population. Distributions of various analyzed metrics were assumed to be normal or Gaussian for simplicity of interpretation. Thus, a Z-score = 1.96 corresponds to 1.96 standard deviations from the mean, which, in terms of Gaussian distribution, corresponds to the 97.5% percentile of the data. Similarly, Z = −1.96 corresponds to the 2.5% percentile. Values of |Z| > 1.96 were considered significantly different from healthy values, whereas positive or negative Z values indicated higher or lower deviations from the healthy control mean values.

As such, it was observed that the patient’s LDF signals deviated significantly from the healthy group, namely regarding endothelial, autonomic and myogenic components in the PORH challenge, especially in the contralateral limb (Figure 3). Also, significant deviations were observed in the healthy group regarding the temperature in the cooling challenge for all components in the ipsilateral limb and cardiac and respiratory components in the contralateral limb. These findings suggest that EM especially impacts the autonomic as well as the myogenic and endothelial microvascular regulation, especially during recovery. In particular, a decreased autonomic response was observed for LDF perfusion in both limbs (Figure 3 top panel), which was accompanied by decreased myogenic and increased endothelial responses in the contralateral limb. At the same time, a decreased endothelial response but increased myogenic and autonomic responses were observed during recovery after the ice cooling challenge (Figure 3 bottom panel). Herein, increased cardiac and respiratory responses were also observed for both limbs. Again, the use of the contralateral limb seems to be informative.

As pointed out above, both global LDF and temperature signals showed a strong positive correlation, particularly for the cooling challenge (rs = 0.820; *p* < 0.001). The inverse relationship observed for the patient between such signal components may suggest that this prior correlation is modified in the case of pathology or otherwise that autonomic and endothelial physiological regulation is compensatory, as could be observed from WT analysis.

After treatment with carvedilol for 30 days, no significant differences in perfusion or temperature were observed between the patient and the healthy group, suggesting a tendency towards normal microvascular function in this patient (Figure 3). These results suggest an interesting clinical application of LDF wavelet analysis to the study of microvascular dysfunction, showing that carvedilol can be used in the treatment of idiopathic erythromelalgia. 

## 5. Conclusions

To the best of our knowledge, this study is the first to apply and investigate the value of WT analysis of LDF signals in the evaluation of microvascular dysfunction. This application to an EM patient allowed a more comprehensive understanding of adaptive mechanisms to known challengers, as compared with a healthy cohort, and suggested a new approach to differentiate specific pathways with clinical interest in the treatment of idiopathic erythromelalgia. This strategy might also be applied to other non-cardioselective beta-blockers, among other drugs. We emphasize that this analysis involving one EM patient, while clearly exploratory, has shown significant deviations in WT-derived physiological components’ values in comparison with the healthy group. The apparent agreement achieved with the clinical evaluation opens the possibility of expanding this approach to other patients and pathologies in vascular medicine as well.

Also noteworthy, is that this study shows that cooling elicits a higher number of measurable changes in LDF components and temperature, especially in higher frequency components (cardiac, respiratory, and myogenic), whereas the PORH especially evokes changes in lower frequency components (myogenic, autonomic, and endothelial), underlying the interest in further exploration of cold temperature as a challenger.

## Figures and Tables

**Table 1:** Characterization of participants. Healthy participants presented as medians and Q1–Q3 (25th empirical quartile to 75th empirical quartile).

	EM Patient	Healthy Participants
N	1	10
Age, years (Q1–Q3)	35.0	27.0 (24.0–30.3)
Body Mass, kg (Q1–Q3)	50.5	59.0 (52.8–64.5)
Height, m (Q1–Q3)	1.6	1.63 (1.60–1.68)
BMI, kg/m^2^ (Q1–Q3)	19.5	21.7 (21.5–23.4)
SYSTP, mmHg (Q1–Q3)	112.0	113.1(105.2–115.1)
DIASP, mmHg (Q1–Q3)	71.0	75.0 (70.0–84.5)
MAP (Q1–Q3)	84.7	87.5 (85.0–94.9)
ABI (Q1–Q3)	1.0	1.1 (1.0–1.1)

BMI, Body Mass Index; SYSTP, Systolic pressure; DIASP, Diastolic Pressure; MAP: Mean Arterial Pressure; ABI, Ankle–Brachial Index.

**Table 2:** Nominal laser Doppler flowmetry and skin temperature values for the healthy control group and the patient, before and after the last treatment (check text); results display variables obtained during the post-occlusive reactive hyperemia and the cooling challenges. Values represent medians and Q1–Q3 (25th empirical quartile to 75th empirical quartile).

		Post-Occlusive Reactive Hyperemia			Cooling		
		Healthy Control	Patient		Healthy Control	Patient	
	Phase	LDF (BPU)	Before	After	LDF (BPU)	Before	After
Ipsilateral	I	27.0; (9.1–49.9)	19.0	9.0	27.5; (11.6–110.5)	19.8	9.1
	II	3.4; (1.2–12.8)	12.8	4.5	11.1; (6.6–92.6)	10.2	3.8
	III	18.9; (14.4–77.0)	8.3	36.4	17.7; (6.5–56.0)	17.7	7.6
	IV	13.5; (8.0–40.4)	10.6	8.1	14.8; (5.8–49.2)	18.5	7.4
Contralateral	I	24.8; (14.5–64.4)	12.6	14.5	34.9; (18.3–108.0)	18.3	7.0
	II	19.0; (12.0–31.4)	4.6	14.1	20.0; (15.1–82.5)	10.9	7.4
	III	20.4; (14.6–56.8)	12.3	13.4	19.7; (14.5–60.1)	10.2	8.8
	IV	16.2; (15.2–35.3)	12.0	8.9	17.8; (12.7–53.8)	11.7	8.9
	Phase	Temp. (°C)	Before	After	Temp. (°C)	Before	After
Ipsilateral	I	23.2; (22.0–26.7)	24.6	21.9	24.4; (22.9–29.7)	24.7	21.8
	II	23.1; (22.1–25.7)	24.4	21.7	19.5; (18.3–24.7)	20.0	15.5
	III	22.9; (22.1–26.5)	24.2	21.7	20.4; (18.3–23.9)	20.6	17.7
	IV	22.8; (22.7–27.3)	24.1	21.7	21.0; (19.2–25.0)	21.8	19.0
Contralateral	I	23.6; (21.3–26.2)	25.0	21.5	23.8; (23.0–30.2)	24.0	21.4
	II	23.4; (21.4–25.7)	24.7	21.4	23.2; (22.1–29.2)	23.8	21.1
	III	23.2; (21.7–26.4)	25.3	21.3	22.8; (21.7–28.6)	23.6	20.8
	IV	23.2; (22.3–25.8)	25.2	21.3	22.8; (21.6–28.2)	23.6	20.7

Phases I, II, III, and IV, respectively, represent baseline rest, challenge (either with post-occlusive reactive hyperemia or ice cooling); early recovery (first 3 min after the challenge), and late recovery (3 to 6 min after the challenge) periods. BPU. Blood Perfusion Units. LDF. Laser Doppler Flowmetry; Temp. Temperature.

## Data Availability

The data presented in this study are available on request from the corresponding author.
